# Investigating the Role π‑Rich Solvents Play in the Growth of Cesium Lead Bromide Nanocrystals

**DOI:** 10.1021/acsnanoscienceau.5c00081

**Published:** 2025-10-24

**Authors:** Tsung-Hsing Chiang, Deborah J. Kerwood, Abigail L. Stapf, Mircea Cotlet, Mathew M. Maye

**Affiliations:** † Department of Chemistry, 2029 Syracuse University, Syracuse, New York 13244, United States; ‡ Center for Functional Nanomaterials, 8099 Brookhaven National Laboratory, Upton, New York 11973, United States

**Keywords:** CsPbBr_3_, perovskite, solvent, hot-injection, plumbate, ^207^Pb NMR

## Abstract

In this report, the role that a high-boiling-point solvent type plays on the nucleation and growth, morphology, and crystal-phase transformation of cesium lead bromide nanocrystals (CsPbBr_3_) is studied. The CsPbBr_3_ products were compared between a one-pot growth mechanism at room temperature (RT) versus a hot-injection mechanism (HI) control using dibenzyl ether (DBE), diphenyl ether (DPE), dioctyl ether (DOE), or 1-octadecene (ODE). The coordination between these solvents and the PbBr_2_ salt precursors resulted in different plumbate [PbSBr_*n*_]^2–*n*^ precursors being formed. The S-to-Pb^2+^ coordination within [PbSBr_*n*_]^2–*n*^ was probed by UV–vis and solvent-phase ^207^Pb NMR, both of which showed considerable coordination between [PbSBr_*n*_]^2–*n*^ and the π-rich DBE and DPE, whose reactivity affected CsPbBr_3_ growth. The effect was more pronounced for CsPbBr_3_ prepared via RT, where the morphology was tunable, with π-rich solvents producing thin rod-like CsPbBr_3_ with a blue emission, compared to the green-emitting thicker platelets formed via HI. While XRD showed crystalline products for both RT and HI, with orthorhombic and cubic forms, respectively, the RT products had considerable surface defects, as was indicated by lower quantum yields, and to understand this the photoluminescent lifetimes were measured by time-correlated single photon counting.

## Graphical abstract

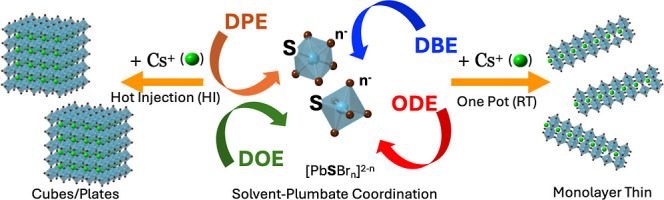

## Introduction

Quantum-confined all-inorganic cesium lead halide nanocrystals CsPbX_3_ (CsPbX_3_, X = Cl, Br, I) are important quantum dots that have potential in light-emitting, energy-harvesting, and sensing applications due to tunable optoelectronics made possible by small changes of stoichiometry, morphology, crystal structure, or combinations thereof.^1–4^ CsPbBr_3_ is the most studied stoichiometry owing in large part to its general stability and visible emission wavelength, and it can be synthesized in both micron-sized single crystals, thin films, and nanosized platelets, cubes, rods, and wires.^5–8^ Moreover, CsPbX_3_ are well known for highly dynamic ionic structures, leading to a variety of crystal types and plausibility of crystal-phase transformation postsynthesis.^6,9–11^ This in particular leads to a wealth of potential postsynthetic treatments via both chemical or physical pathways,^12,13^ tuning the dimensionality of the crystals.^14–19^ Such conditions include ligand functionality, solvent polarity, and reaction temperature.^7,20–24^ Recently, it has also been shown that synthetic variability can lead to transformations from CsPbX_3_ into Cs_4_PbX_6_, and quasi-2D CsPb_2_X_5_, which allow a pathway for further optoelectronic and function tunability.^25–29^

Changes in growth mechanism also control of CsPbBr_3_ growth, such as in the use of hot injection,^1,30^ microwave irradiation,^31–33^ solvothermal,^34,35^ ultrasonication,^36–38^ ligand assistance,^39,40^ and even mechanochemistry.^41–43^ The role of precursors has also been explored, especially organohalide sources, with benzoyl bromide,^30^ 1-bromohexane,^44^ bromobenzene,^45^ 2-bromododecanoic acid,^11^ α-haloketones,^46^ and silylhalides.^47^ With each showing the ability to tailor complexation with lead, this leads to controlled CsPbBr_3_ growth. Other routes employ ligands, like oleylamine (OAm) and oleic acid (OAc) to digest PbX_2_ salts, creating mixtures of metal oleate and ammonium halide before growth.^48–50^ One interesting phenomena of CsPbX_3_ growth using each precursor is that nucleation, even in a highly coordinating environment, has been shown to occur rapidly at low temperatures.^51,52^ This has been used to understand growth kinetics and pathways, via intentionally slowing the reaction for instance,^44,53^ as well as to employ stop-flow spectroscopy^54,55^ and in situ X-ray scattering.^56^ Moreover, machine learning has also used such kinetic growth information for insights int nanosheet growth.^55^ Thus, the room-temperature approaches are a pathway toward fabricating such important materials at low cost, using flow chemistry for example,^57,58^ or other high-throughput methods.^59^

Therefore, understanding the role that each component plays in growth is important, especially from the solvent perspective. In addition to the halide sources mentioned above, studies have also focused on the ligand composition, such as secondary amines,^60^ as well as chain lengths of both acids and amines.^21^ The reason why all of these studies are so important is the interesting fact that there are so many different Pb^2+^ coordination complexes, or plumbates ([PbBr_*n*_]^2–*n*^), that are possible during precursor formation and crystal growth, as are the formation of Pb_*x*_Br_*y*_^2*x*–*y*^ polyhedra, L_2_[PbBr_4_] nanosheets and magic-sized clusters,^54,55^ and even CsBr nuclei intermediates.^44,45^ In addition, solvent polarity and basicity has also been explored.^61–64^ Moreover, solvents with different characteristics create unique combinations of [PbBr_*n*_]^2–*n*^, altering growth.^12^ We recently explored the role that high-boiling-point solvents play in the formation of [PbBr_*n*_]^2–*n*^ using a MWI method^33^ and that study led us to hypothesize how different high-boiling-point solvents may be coordinating to the [PbBr_*n*_]^2–*n*^ precursors and allow for the further tailoring of CsPbBr_3_ growth.

Herein, we investigate that hypothesis by monitoring the formation of [PbBr_*n*_]^2–*n*^ in the presence of π-rich high-boiling-point solvents and ligands. The [PbBr_*n*_]^2–*n*^ formation and stability is first measured and characterized and then used in the growth of CsPbBr_3_ at room temperature (RT) using a one-pot growth mechanism (RT). These results are then compared to a control study of CsPbBr_3_ growth at high temperatures using a hot-injection (HI) mechanism, and the differing nanoparticle products are compared.

## Materials and Methods

### Chemicals

Cesium carbonate (Cs_2_CO_3,_ 99%), lead­(II) bromide (PbBr_2,_ 99.999%), lead­(II) nitrate (Pb­(NO_3_)_2_, 99.99%), 1-octadecene (ODE, 90%), oleylamine (OAm, 70%), oleic acid (OAc, 90%), dibenzyl ether (DBE, 98%), diphenyl ether (DPE, ≥99%), dioctylether (DOE, 99%), toluene (Tl, >99%), and tetraoctylammonium bromide (TOABr, 98%) were purchased from Sigma-Aldrich and used as received. Dimethylformamide (DMF, ≥99.8%) was purchased from Fisher scientific and used as received. Hydrogen bromide (HBr, 49%) was purchased from Alfa Aesar. The deuterated dimethyl sulfoxide (DMSO-*d*_6_, 99.9%), and deuterium oxide (D_2_O, 99.9%) was purchased from Cambridge Isotope Laboratories and used as received.

### Cesium Precursor Preparation

In a typical reaction, cesium oleate (CsOAc) was first prepared for both RT and HI synthesis. The RT precursor was prepared by combining 0.163 g of Cs_2_CO_3_ (0.5 mmol), 1.58 mL of OAc (5 mmol), and 3.42 mL of ODE (10.69 mmol) in a three-neck flask. The HI precursor was prepared by combining 0.27 g of Cs_2_CO_3_ (0.83 mmol), 0.83 mL of OAc (2.6 mmol), and 10 mL of ODE (31.25 mmol). These mixtures were first heated to 120 °C under vacuum until all Cs_2_CO_3_ dissolved, and no gas evolution remained. The final solution was transparent with a yellow color and was stored under N_2_. During storage, the CsOAc solution could solidify and was warmed to 50–80 °C before use. The ODE was replaced by DBE, DOE or DPE for the different solvent trials.

### One-Pot Growth at Room Temperature

In a typical RT synthesis, PbBr_2_ (0.069 g, 1.88 mmol), ODE (5 mL, 15.63 mmol), OAc (0.5 mL, 1.58 mmol), and OAm (0.5 mL, 1.52 mmol) were placed into a 25 mL glass vial, purged, and sealed with N_2_ and heated to 100 °C while stirring. Once the PbBr_2_ dissolved, the solution was cooled to room temperature, and then, 0.4 mL of the CsOAc precursor prepared above was injected swiftly into the vial. Aliquots were taken for characterization at different reaction times. The ODE was replaced by DBE, DOE or DPE for different solvent trials. The crude CsPbBr_3_ solution was then transferred to a series of 1.5 mL centrifuge tubes and centrifuged at 10,000 rpm for 30 min. The supernatant was discarded, and the precipitant product was redispersed using toluene. This CsPbBr_3_ solution was then centrifuged a second time at 6000 rpm to remove any large insoluble byproducts.

### Hot Injection Nucleation and Growth (HI)

In a typical HI synthesis, PbBr_2_ (0.069 g, 1.88 mmol) and ODE (5.0 mL, 15.63 mmol) were placed into a three-neck flask, and the solution was heated to 120 °C under vacuum for 1 h to degas. Next, the synthesis flask was switched from vacuum to N_2_ and the solution was heated to 140 °C. 0.5 mL portion of OAm (1.57 mmol) and 0.5 mL of OAc (1.52 mmol) were added sequentially to dissolve PbBr_2_. In a separate bottle or flask, the CsOAc precursor prepared above was heated to 80 °C. Next, 0.4 mL of CsOAc was quickly injected into the flask, upon which the heating mantle was removed to quench the reaction or left to heat depending on the desired annealing time. ODE was replaced by DBE, DOE, or DPE for the different solvent trials. The crude CsPbBr_3_ solution was then purified in the same manner as for the RT products above.

### Plumbate Preparation and Analysis Benesi–Hildebrand Analysis

Two different plumbate precursors were prepared for optical measurements, one with and one without additional ligands. In the first, PbBr_2_ (0.069 g, 1.88 mmol), 5 mL solvent (ODE, DBE, DOE, or DPE), OAc (0.5 mL), and OAm (0.5 mL) were placed into a round-bottom flask and heated to 120 °C under vacuum for an hour to dissolve the PbBr_2_. The solution was then cooled and stored in a glass vial. In the second, PbBr_2_ (0.03 mmol) was dissolved in 5 mL of the solvent without OAc or OAm. Due to the lower solubility, the solution was first heated to 120 °C under vacuum for an hour and then stirred and heated at 120 °C overnight under an Ar gas before being cooled and stored. Control plumbates for the BH analysis were formed by creating a 0.4 mM PbBr_2_ stock solution dissolved in DMF, which formed a soluble plumbate, which we denote [Pb­(DMF)_6_]^2+^ for simplicity, but the two Br^–^ counterions exchange with DMF. In a typical BH analysis, the control plumbates, either [PbSBr_*n*_]^2–*n*^ where S = ODE, DBE, DPE, DOE, or [Pb­(DMF)_6_]^2+^, were titrated with Br^–^ from a 50 mM TOABr stock solution using the same solvent as the plumbate. Special care was used in the case of the case subtracting the baseline absorption from the solvents because they absorb strongly below ∼375 nm (See Figure S1), and a double-beam UV–vis setup was used throughout.

### Solution-Phase ^207^Pb NMR

NMR experiments were performed using samples that originated from a 1.0 M PbBr_2_ solution dissolved readily in deuterated DMSO (DMSO-*d*_6_). The resulting plumbate, which we denote [Pb­(DMSO-*d*_6_)_6_]^2+^ for simplicity, but the two Br^–^ counterions coordinate were found in NMR to strongly coordinate to Pb^2+^, see below. To a [Pb­(DMSO-*d*_6_)_6_]^2+^ aliquot (0.6–1.0 mL), a series of volume additions of ODE, DOE, DPE, DBE, and OAc/OAm (1:1) were studied. To better understand and tabulate the ^207^Pb chemical shift (δ), several different control plumbates were also formed. For this, a 1.0 M solution of Pb­(NO_3_)_2_ was prepared in DMSO-*d*_6_, forming a bromine free and soluble [Pb­(DMSO-*d*_6_)_6_]­(NO_3_)_2_ plumbate. The significant difference in the ^207^Pb chemical shift between [Pb­(DMSO-*d*_6_)_6_]­(NO_3_)_2_ (δ = −2635 ppm) and the [Pb­(DMSO-*d*_6_)_6_]­Br_2_ described above (δ = −156 ppm) demonstrates the coordination of Br to Pb. Next, aqueous aliquots of HBr were added to volumes of [Pb­(DMSO-*d*_6_)_6_] (0.6–1.0 mL) at 0.25, 0.5, 1.0, 2.0, 4.0, and 6.0 mol equiv ([Br^–^]/[Pb^2+^]).

### Instrumentation

The ultraviolet–visible (UV–vis) spectra were acquired on Cary 50 or Cary 100 UV–vis spectrophotometers (Agilent Inc.). The photoluminescence (PL) measurements were performed on a Cary Eclipse spectrophotometer (Agilent Inc.) using an excitation wavelength of 365 nm. A quartz cuvette with a 1 cm path length was used for both UV–vis and PL characterizations. The scanning electron microscopy (SEM) results were collected on a JSM-IT100 instrument (Jeol Inc.) with sample drop cast onto freshly cleaved HOPG substrates, while the transmission electron (TEM) micrographs were taken on a JEOL-1200 (JEOL Inc.) using carbon-coated grids. The SEM were performed at SUNY-ESF, while the TEM measurements were collected at SUNY-Upstate Medical School. A D2 PHASER X-ray diffractometer (Bruker Inc.) was used for XRD with samples either drop cast from dispersed solutions and dried onto zero diffraction silicon substrates or by the addition of dried powders to the substrates. Diffraction patterns were compared to reference patterns provided by the crystallography Open Database (COD) and fitted via DIFFRAC.EVA software (Bruker Inc.). The NMR experiments were performed on an AVANCE III HD spectrometer operating at 400 MHz (Bruker Inc.). The ^207^Pb measurements were performed by first calibrating the ^207^Pb chemical shift of Pb­(NO_3_)_2_ dissolved in D_2_O to δ = −2961 ppm and then the samples were measured with a frequency of 87.7 MHz, a relaxation delay of 2 s, and an acquisition time of 0.20 s by a room-temperature BBO probe at 300.0 K.^65^ Time-resolved PL decay measurements were performed with a Fluotime FT200 time correlated single photon counting (TCSPC) instrument (Picoquant Inc.) using a 442 nm pulsed diode laser as an excitation source. The overall response function of this instrument is 25 ps, and the decays were collected with a PicoHarp 300 data analyzer and fit with Fluofit software (Picoquant Inc.) using exponential models. The TCSPC measurements made use of the Advanced Optical Facility at Brookhaven National Laboratory.

## Results and Discussion

In this section, the role of the solvent (S) type on the formation of solvent-containing plumbate precursors, denoted [PbSBr_*n*_]^2–*n*^, is first probed by UV–vis and then by solution-phase ^207^Pb NMR. The [PbSBr_*n*_]^2–*n*^ are then used as precursors in the synthesis of CsPbBr_3_ using two different growth mechanisms, a one-pot method at room temperature, denoted RT, and a nucleation and growth mechanism via hot-injection at 140 °C, denoted HI. The resulting optoelectronic properties, crystallinity, and morphology are then studied via UV–vis, PL, XRD, TEM, and SEM.

The strategy for precursor formation is shown in Figure 1a. Lead bromide (PbBr_2_) salt, which has an isomorphous trigonal prismatic crystal structure where lead Pb^2+^ is surrounded by nine Br^–^, can be dissolved in the presence of Lewis bases (ligands, L), coordinating solvents (S), or combinations thereof. Depending on the coordination, monomeric plumbates like [PbBr_*n*_]^2–*n*^ can be formed, as can polymeric, corner, or edge-sharing polyhedra, like [Pb_*x*_Br_*y*_]^2*x*−*y*^, the charge of which is balanced via counterions, or in this case, via L or S. with varied halide stoichiometries and complex charges, which are then used as precursors for CsPbBr_3_. To differentiate which [PbBr_*n*_]^2–*n*^ is formed, the characteristic UV–vis absorption is measured, which results from both metal sp transition and ligand–metal charge transfer (LMCT).^66^

**Figure 1:**
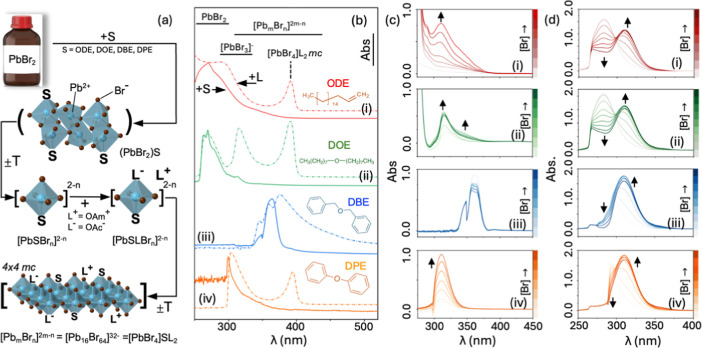
(a) Idealized schematic of [PbSBr_*n*_]^2–*n*^ formation, where solvent (S) is first added to PbBr_2_ followed by heating (T) and addition of ligand (L), that form S- or L-stabilized plumbate monomers ([PbSBr_*n*_]^2–*n*^) that form larger [Pb_*m*_Br_*n*_]^2*m*−*n*^ polyhedral, like a 4 × 4 magic-sized cluster^REF^.^54,55^ (b) Normalized UV–vis of [PbSBr_*n*_]^2–*n*^ prepared in S = ODE (i), DOE (ii), DBE (iii), and DPE (iv) without additional L (solid lines), and with addition L = OAm + OAc (dashed lines). The change in the UV–vis signature of [PbSBr_*n*_]^2–*n*^ in pure S = ODE (i), DOE (ii), DBE (iii), and DPE (iv) upon addition of TOABr. (d) Change in UV–vis of [Pb­(DMF)_6_]^2+^ controls in S = ODE (i), DOE (i), DBE (iii), and DPE (iv) upon the addition of TOABr. See Figure S2 for additional details.

For example, the solid lines in Figure 1b show the UV–vis of [PbSBr_*n*_]^2–*n*^ formed in this study by complexation with S = ODE (i), DOE (ii), DBE (iii), and DPE (DPE) in the pure solvent after heating and sonication (see methods). Previous studies of plumbates have assigned approximate absorption ranges of specific plumbates,^67^ where PbBr_2_, [PbBr_3_]^−^, and [PbBr_4_]^2–^ at 260, 310, and 360 nm in DMF, respectively.^66^ In our study, the PbBr_2_ salts have limited solubility in ODE and DOE, with only absorption at <300 nm, which has been associated with exfoliated solid PbBr_2_ or PbBr_2_ nanoparticles. In contrast, complexation with the π-rich DBE and DPE results in red-shifted spectra, especially for DBE, whose energy suggests solubilized higher order [Pb_*x*_Br_*y*_]^2*x*−*y*^. Here, we note that because both BE and DPE absorb strongly in the UV region, any absorptions in those regions are not observed. The transmittance measurements for each S are shown in Figure S1. As expected, the addition of the ligands (L = OAc + OAm (1:1)) greatly aids dissolution, as shown by the dashed lines in Figure 1b. For example, the formation of [PbSBr_*n*_]^2–*n*^ with a higher complexity is clearly assisted with the presence of L, and new absorption bands are observed in the ODE, DOE, and DPE, each residing at ∼370 nm. Recent studies have demonstrated that the band corresponds with a [PbBr_4_]­L_2_ complex, which is a magic-sized cluster (mc), a 4 × 4 corner shared polyhedra sheet for instance.^54,55^ Interestingly, compared to the other solvents, the plumbate containing DBE shows absorption from 425 to 475 nm, which suggests further coordination by DBE after [PbBr_4_]­L_2_ formation and potentially larger cluster sizes. Here, we note that in order to achieve the PbBr_4_ stoichiometry, excess Pb^2+^ either must remain in solution, been purified, or reside as unsaturated cations or counterions, unlike the studies mentioned above, which used benzoyl bromide to tailor precise Pb/Br stoichiometry.^55^

These results suggest that the π-rich DPE and DBE bind [PbSBr_*n*_]^2–*n*^ more strongly than ODE and DOE. We quantitatively probed this by using a competitive assay where the [PbSBr_*n*_]^2–*n*^ precursors were first formed and then Br^–^ from a source other than PbBr_2_ was added. Thus, higher order (*n*) plumbates could then be formed, of which the occurrence can be observed optically. Figure 1c,d shows two such studies, in a so-called Benesi–Hildebrand (BH) competitive assay.^7,68,69^ In Figure 1c, the [PbSBr_*n*_]^2–*n*^ formed above were reacted with tetraoctylammonium bromide (TOABr) as the Br^–^ source, and the rise in the intensity of red-shifted absorption bands is a semiquantitative way to monitor Br^–^ complexation. The results indicated that [PbBr_4_]^2–^ is formed in DPE and ODE while adding excess Br^–^, with higher calculated Ka of both [PbBr_3_]^−^ and [PbBr_4_]^2–^ from DPE demonstrate that lead plumbates are favorable to form in DPE than in ODE (Figure S2). In DBE, decreased absorbance is observed, suggesting conversion from [PbBr_4_]^2–^ to higher level plumbates like [PbBr_5_]^3–^ or [PbBr_6_]^4–^.

To better understand these results, control experiments were performed using a starting plumbate that did not have a strong absorption in the region of interest. This was achieved by forming [Pb­(DMF)_6_]^2+^ via dissolving PbBr_2_ with DMF, a strongly coordinating solvent that has been studied previously in BH analysis.^70^ In Figure 1d, the [Pb­(DMF)_6_]^2+^ was first reacted with S, followed by titration with TOABr.^71^ Thus, when compared to [Pb­(DMF)_6_]^2+^ alone, any changes to the BH slope are indicative of competing binding by S and thus serve a proxy for inhibition. For instance, the results indicate that [PbBr_3_]^−^ formation is similar for both BE (*K*_a_ = 0.520 mM^–1^) and DPE (*K*_a_ = 0.545 mM^–1^), compared to DOE (*K*_a_ = 0.308 mM^–1^) and ODE (*K*_a_ = 0.297 mM^–1^). For [PbBr_4_]^2–^, the *K*_a_ again trended closely with π-density, with BE (*K*_a_ = 0.047 mM^–1^) and DPE (*K*_a_ = 0.050 mM^–1^) being higher than those of the ODE (*K*_a_ = 0.005 mM^–1^). However, OE (*K*_a_ = 0.072 mM^–1^) is slightly higher than that of BE and DPE.

To further probe S-to-Pb^2+^ coordination in these [PbSBr_*n*_]^2–*n*^ precursors, solution-phase ^207^Pb nuclear magnetic resonance (NMR) was employed.^72^ This approach is similar to using ^119^Sn NMR, which was recently used to study precursor formation in tin halide perovskites.^73^ The ^207^Pb isotope is sensitive to local dielectric or electronic changes, has a wide chemical shift range (Δδ ∼ 16,000 ppm), and has been used previously to study plumbates.^65,74,75^ Similar to the BH analysis above, a model plumbate was used, in this case [Pb­(DMSO-*d*_6_)_6_]^2+^ formed by dissolving the PbBr_2_ salt with DMSO-*d*_6_,^76^ which was then titrated with S in the same manner as the UV–vis.

Figure 2 shows the ^207^Pb NMR spectra corresponding to reacting [Pb­(DMSO-*d*_6_)_6_]^2+^ with increasing concentrations of S = ODE (i), DBE (ii), DOE (iii), and DPE (iv). The [Pb­(DMSO-*d*_6_)_6_]^2+^ has a δ = −184 ppm, which corresponds closely to a control plumbate with 2 equiv of Br^–^ added (see Figure S5). There is a negligible δ shift (Δδ) when ODE or DOE are added, while on the other hand, the addition of DBE and DPE induce significant positive Δδ with increasing [S].^77^ These positive Δδ indicate a strong coordination by DBE and DPE, as confirmed with a HBr titration of a control plumbate (see Figure S5) because those plumbates are deshielded due to various basicity from DMSO. For example, DBE has a Gutmann’s donor number (DN) of 19.0, and DMSO has a DN at 29.8,^78^ suggesting DMSO is the stronger Lewis base. The addition of ligands to the S coordinate [Pb­(DMSO-*d*_6_)_6_]^2+^ induces a further positive shift, as shown in (v), where OAm/OAc are added to the DPE system at 0.5–2.0 equiv, suggesting further coordination to Pb^2+^, or the ligands acting as counterions to the plumbate. This counterion affect was also confirmed in a control experiment using [PbBr_4_]^2–^ that was reacted with an additional 2 equiv of OctAm, which caused a Δδ of +24 ppm (see Figure S6). When the OAm/OAc is added directly to the [Pb­(DMSO-*d*_6_)_6_]^2+^, a positive Δδ ∼ 60 ppm is observed. The inset in Figure 2b plots the Δδ relationship with molar equivalents ([S/L]/[Pb^2+^]).

**Figure 2:**
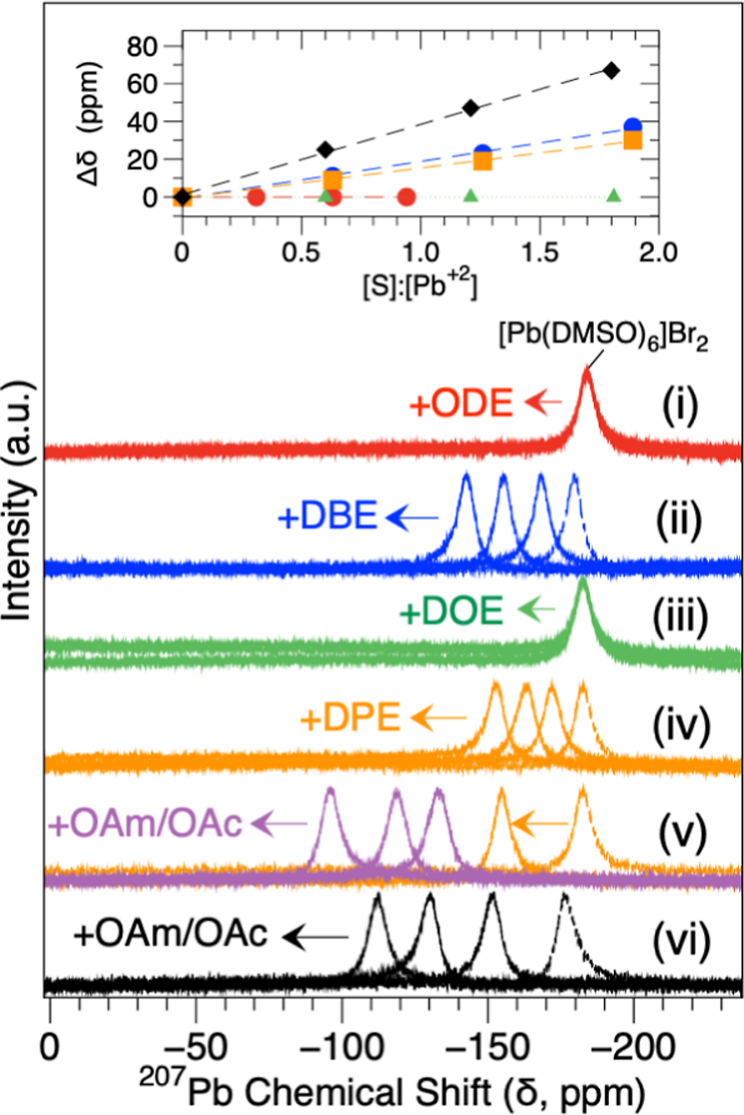
Representative liquid-phase ^207^Pb NMR of [Pb­(DMSO-*d*_6_)_6_]^2+^ upon the addition of increasing molar equivalents ([S]/[Pb^2+^]) of ODE (i), DBE (ii), DOE (iii), DPE (iv), the addition of OAm + OAc to DPE (v), and only the addition of OAm + OAc (1:1) (vi). Inset: A plot of chemical shift change (Δδ) versus molar equivalents added. Each sample contained 0.2 M [Pb­(DMSO-*d*_6_)_6_]^2+^ stock solution in DMSO-*d*_6_ that originated from PbBr_2_ and was reacted for ∼12 h.

Next, these [PbSBr_*n*_]^2–*n*^ ions were used as precursors in the synthesis of CsPbBr_3_. Figure 3a shows an idealized schematic of a one-pot room temperature (RT) synthesis. Briefly, [PbSBr_*n*_]^2–*n*^ was added to a synthetic flask containing excess S (OAc/OAm = 1:1). Next, a previously prepared solution of cesium oleate (CsOAc) was injected swiftly, upon which the solution changed color from translucent yellow to turbid blueish green immediately. The reactions were then annealed at room temperature, and sampled over reaction times (*t*) of 1–96 h to observe UV–vis or PL changes.

**Figure 3:**
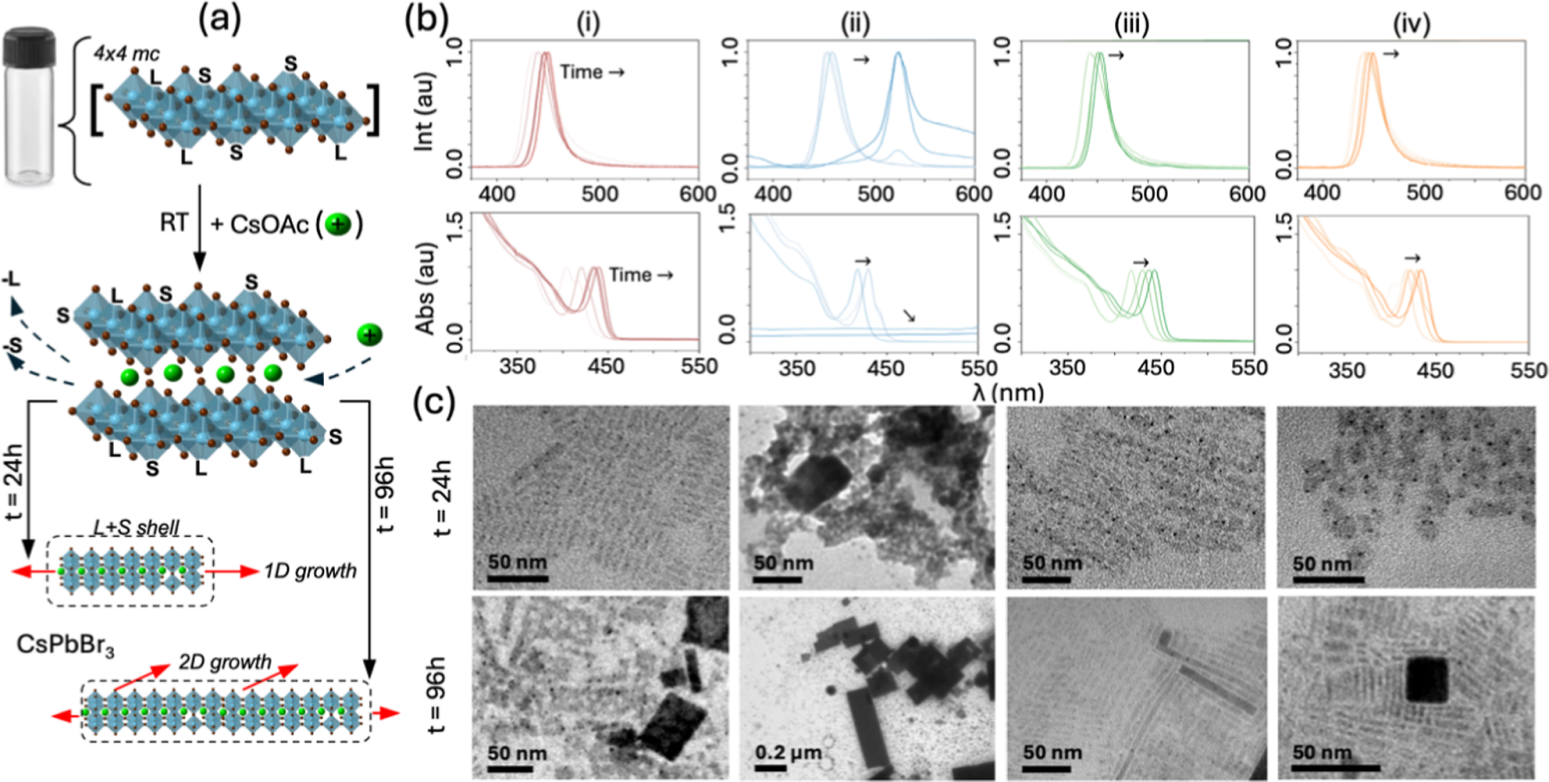
(a) Schematic illustration of room-temperature (RT) one-pot growth of RT-CsPbBr_3_, where different [PbSBr_*n*_]^2–*n*^ are formed in S as described above, and reacted with CsOAc, which assembles the plumbates or polyhedral clusters in 1D over reaction times of *t* = 24 h, and then more 2D growth at longer times. (b) Representative PL emission (top) and UV–vis absorption (bottom) of CsPbBr_3_ from [PbSBr_*n*_]^2–*n*^ formed in S = ODE (i), DBE (ii), DOE (iii), and DPE (iv), over the course of a reaction from *t* = 1–96 h. (c) TEM micrographs of RT-CsPbBr_3_ products after *t* = 24 h (top) and *t* = 96 h (bottom). See enlarged TEMs and size distribution histograms in Figures S7 and S8.

Figure 3b shows the observed optoelectronic properties. The primary photoluminescent (PL) peak maxima (λ_PL_) measured were ∼438 (ODE), ∼453 (DBE), ∼443 (DOE), and ∼440 nm (DPE), respectively, at ∼1 min. Of interest was that samples from DOE, ODE, and DPE showed red shifts of only ∼10 nm after 96 h, while samples from DBE consistently showed large shifts of up to 80 nm. The PL from DOE, ODE, and DPE were narrow, with full width half maxima (fwhm) of 17–21 nm, while the fwhm of DBE was broader at ∼50 nm, suggesting crystal growth is inhibited and final products more polydisperse, see below. The first band edge absorption (λ_Abs_) was measured by UV–vis, as also shown in Figure 3b. The λ_Abs_ is known to be a function of the CsPbBr_3_ thickness or the number of unit-cell monolayers (ML), where each ML is defined as a corner-sharing [PbBr_6_]^4–^ (0.59 nm). The observed λ_Abs_ of ∼445 nm imply ∼2.5 MLs or ∼1.5 nm thick CsPbBr_3_ products.^61^ Of interest are the DBE-CsPbBr_3_ at *t* = 24 and 96 h, which were turbid and absorbance appear flat due to the scattering of the sample, suggesting either aggregations or a large CsPbBr_3_ morphology.^79^

Such morphology differences of the RT-CsPbBr_3_ products were imaged via Transmission Electron Microscopy (TEM), as shown in Figure 3c for samples at *t* = 24 (top) and 48 h (bottom). At 24 h, the ODE-CsPbBr_3_ are short rod-like platelets with an average length (*l*) of ∼16 nm, the DOE-CsPbBr_3_ are wire like with *l* ∼ 31 nm length, while the DPE-CsPbBr_3_ are platelets with *l* ∼ 24 nm. Again, the DBE-CsPbBr_3_ is an outlier, with irregular aggregates and small clusters. After 96 h, the ODE-CsPbBr_3_ have lengthened to *l* ∼ 28 nm, while both DOE- and DPE-CsPbBr_3_ both transformed into more plate-like structures, wider and longer. The DBE-CsPbBr_3_ still contains small amorphous clusters, but large mesoscale plates with *l* ∼ 2.5 μm were also a primary product. It is interesting to note what our previous studies using BE also showed populations of these large plates.^33^

Next, the crystal structure was compared by using powder X-ray diffraction (XRD). Figure 4a compares the results of the ODE- (i), DBE- (ii), DOE- (iii), and DPE-CsPbBr_3_ (iv) prepared at RT at *t* = 24. The observed diffraction pattern for ODE-, DOE-, and DPE-CsPbBr_3_ suggest a CsPbBr_3_ crystalline product, which is more highly concentrated with orthorhombic phases than cubic ones, when compared to those standards. This is especially true when considering the splitting observed at 2θ = 30.25° and 30.54°. As suggested by the optoelectronic properties above, the BE-CsPbBr_3_ is also an outlier in the crystal structure, where mixed phases that index with CsPbBr_3_, Cs_4_PbBr_6_, and Cs_2_PbBr_5_ as well as PbBr_2_ are observed, suggesting that those phases may be the primary product or are challenging to remove during purification. The overall sharpness of the diffraction and narrow width half-maximum (fwhm) of 0.4–0.6° suggest uniform crystal growth.

**Figure 4:**
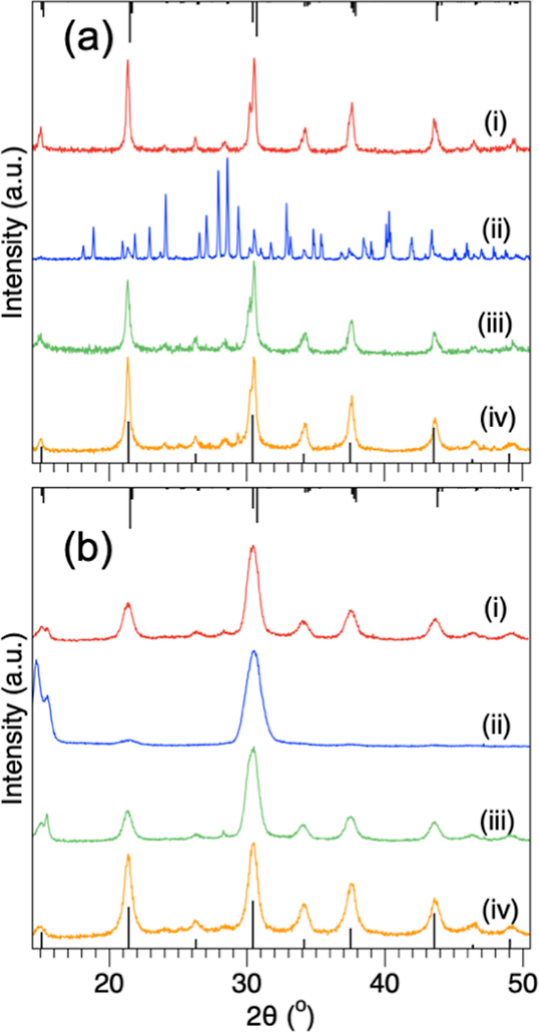
Powder XRD of CsPbBr_3_ prepared via RT at *t* = 24 (a) and HI at *t* < 1 min (b) using ODE (i), DBE (ii), DOE (iii), and DPE (iv). Comparison CsPbBr_3_ reference patterns for the cubic (bottom, COD-1533063) and orthorhombic (top, COD-4510745) shown.

Taken together, these results demonstrate that these solvents do influence the growth characteristics and the final properties of the CsPbBr_3_ when prepared at room temperature and that the [PbSBr_*n*_]^2–*n*^ plumbates formed via DBE and DPE have the strongest coordination, as shown by both UV–vis and NMR studies, and by the difficulty in forming a well-defined and crystalline BE-CsPbBr_3_ product. We hypothesized that at elevated temperatures, the more stable DBE plumbate could still be used to prepare crystalline CsPbBr_3_, and that other solvent effects would be minimized. To test this, the same set of [PbSBr_*n*_]^2–*n*^ precursors were used in the nucleation and growth of CsPbBr_3_ via the hot injection method (HI).

Figure 5a shows an illustration of the synthesis. Clearly, the biggest difference in the mechanism is the effect of temperature, which likely digests [PbSBr_*n*_]^2–*n*^ into more monomeric forms at *T* = 140 °C, before the injection of CsOAc. In this study, the reaction was left to proceed for *t* < 1 min and *t* = 30 min. The optoelectronic properties are shown in Figure 5b for ODE- (i), DBE- (ii), DOE- (iii), and DPE-CsPbBr_3_ (iv) at *t* < 1 min (top) and *t* = 30 min (bottom). Unlike the products formed above via RT, each product showed a similar absorption and PL profiles at *t* < 1 min, with a UV–vis λ_Abs_ of 480–512 nm, suggesting a thickness of 5–20 ML, and a corresponding PL of λ_PL_ of 508–518 nm. After the reaction was proceeded at a temperature for 30 min, some aggregation was observed, especially for the ODE-CsPbBr_3_ product.

**Figure 5:**
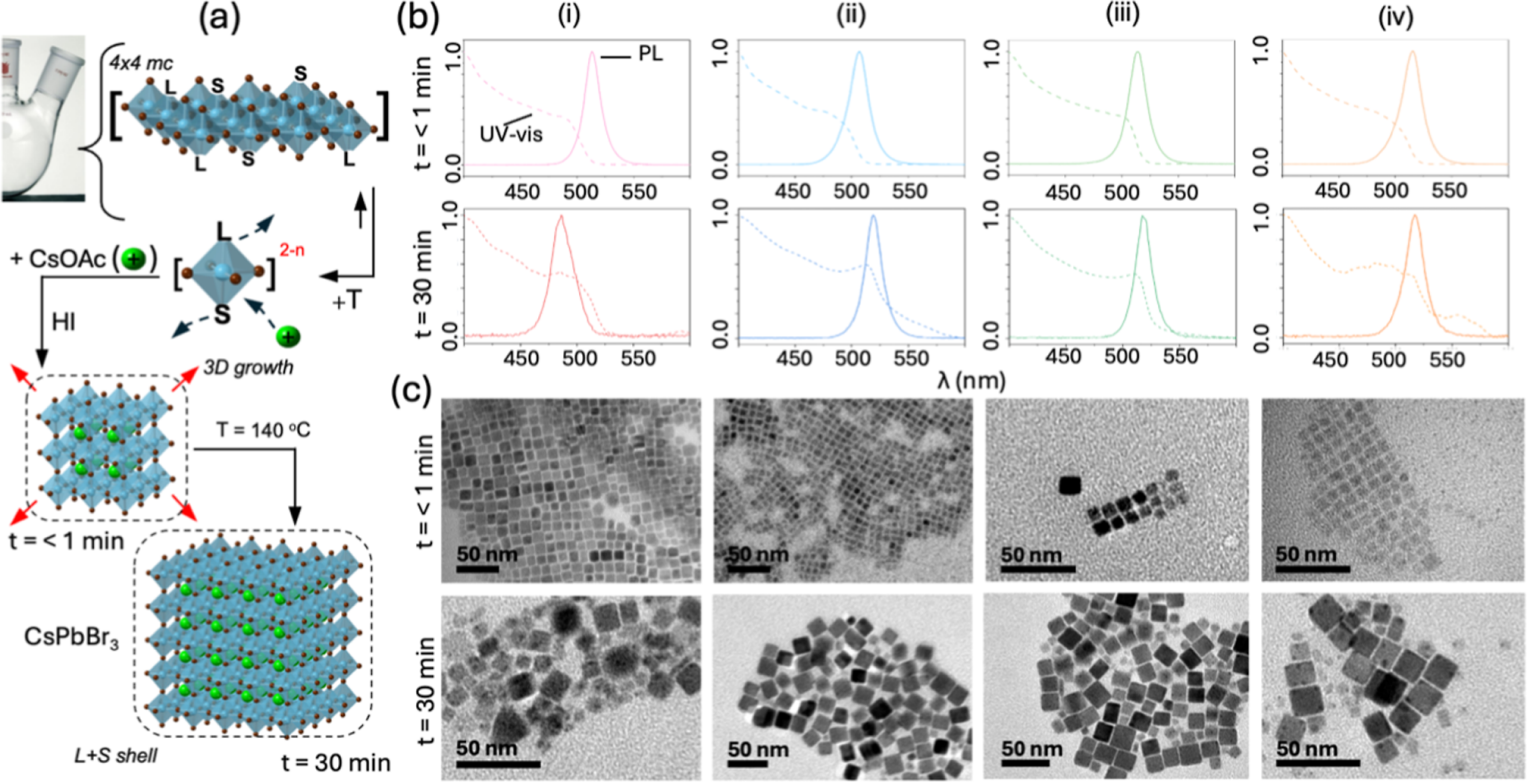
(a) Schematic of hot-injection (HI) growth. Using the different [PbSBr_*n*_]^2–*n*^ plumbates prepared above, the system is heated to *T* = 140 °C and CsOAc is added, digesting the larger plumbate into monomers which then grow in a 2D and then 3D manner at *t* = 1–30 min. (b) Overlayed UV–vis absorption and PL emission plots for S = ODE (i), DBE (ii), DOE (iii), and DPE (iv) sampled at reaction times of *t* < 1 min (top), and *t* = 30 min (bottom). (c) TEM micrographs of the same HI-CsPbBr_3_ after *t* < 1 min (top) and *t* = 30 min (bottom) for same samples shown in c. See enlarged TEMs and size distribution histograms in Figures S9 and S10.

The top panel of Figure 5c shows TEM micrographs for the HI CsPbBr_3_ products *t* < 1 min, where small thin 2D CsPbBr3 are observed, with *l* = 6–8 nm (Figure S6). At *t* = 30 min, the particles were shown to have grown uniformly into cubes with *l* between 8 and 16 nm. These sizes and morphologies are in contrast to the RT-CsPbBr_3_ products shown above, and no wires or thin rods were observed. In addition, the BE products show crystalline and well-defined CsPbBr_3_ with a lack of large plates, unlike the RT BE products shown above which were amorphous at short reaction times but formed large plates at longer ones.

Figure 4b shows the XRD of the HI-CsPbBr_3_ products using ODE (i), DBE (ii), DOE (iii), and DPE (iv). Compared to the RT-CsPbBr_3_, each product, including the BE-CsPbBr_3_ shows a strong diffraction that also indexes to CsPbBr_3_. Two differences are observed, however. First, the single diffraction at 2θ = 30.5° more closely aligns with the cubic crystal structure because it lacks the splitting at 2θ = 30.25° and 30.54°. Second, and interestingly, the fwhm of the diffraction are larger (∼1°) than the RT products (0.4–0.6°), despite the particles themselves being both larger and more three-dimensional. This suggests that the individual HI-CsPbBr_3_ may be more polycrystalline and may also have regions of the orthorhombic phase.

Because of the similarity of the optoelectronic, morphological, and crystallinity properties of the HI-CsPbBr_3_, it is clear that those products are similar and that the solvent effect on [PbSBr_*n*_]^2–*n*^ formation and stability is minimized at elevated temperatures. This demonstrates that the RT route is beneficial over HI when it comes to the synthesis of both thin CsPbBr_3_ nanoparticles and also a rod-like morphology. Because of this, we speculate that the further tailoring of the [PbSBr_*n*_]^2–*n*^ reactivity could have a profound effect for future synthesis designs. However, one limitation of the RT method was the overall lower quantum yields observed, despite good monodispersity in size, shape, and crystallinity of products. We largely attributed this to surface defects or more likely surface bound and unsaturated plumbates.

To probe surface defects and trap sites, we characterized the CsPbBr_3_ via time correlated single photon counting (TCSPC) because the PL lifetime (τ) is an indicator of crystallinity and surface defects. Figure 6 shows the TCSPC comparisons for the ODE- (a), DBE- (b), DOE- (c), and DPE-CsPbBr_3_ (d) products prepared via both RT and HI methods. As mentioned above, quantum dot lifetimes are dependent upon a number of factors, ranging from the composition, size, shape, crystallinity, and surface defect trapping sites,^80–83^ and a number of studies have focused on the PL decay of CsPbBr_3_, including the role that short-chain acids and other small molecules can play.^80,84,85^ One consistent observation from our measurements was the longer τ for the HI products compared to those from RT, which in general were mono exponential. However, to best compare the τ across the samples, the PL-decay were fit to a biexponential decay function, which characterized a fast component (τ_1_) for decays <5 ns, and a long component (τ_2_) for decays >5 ns, which could then be combined into an amplitude-weighted average lifetime (τ_Ave_). These results are shown in Table S1. For instance, the ODE-CsPbBr_3_ prepared via HI and shown in Figure 6a had a τ_Ave_ of 8.53 ns and a single monomodal decay, while the RT product had a τ_Ave_ of 2.23 ns and a bimodal decay, which had a strong τ_1_ value of ∼1 ns, which is often attributed to ultrafast recombination sites at the surface. The DBE-CsPbBr_3_ showed the largest variation, with the HI product has τ_Ave_ ∼ 43.62 nm compared to a τ_Ave_ ∼ 1.66 ns for the RT product. This sample in particular highlights the effect of S coordination, which for HI results in thick platelet products but at RT highly defective semiamorphous crystallites. This is futher evidence that that unreacted [PbSBr_*n*_]^2–*n*^ reside at the surface of the CsPbBr_3_ formed via RT, further contributing to the poor growth and adding a recombination site.

**Figure 6:**
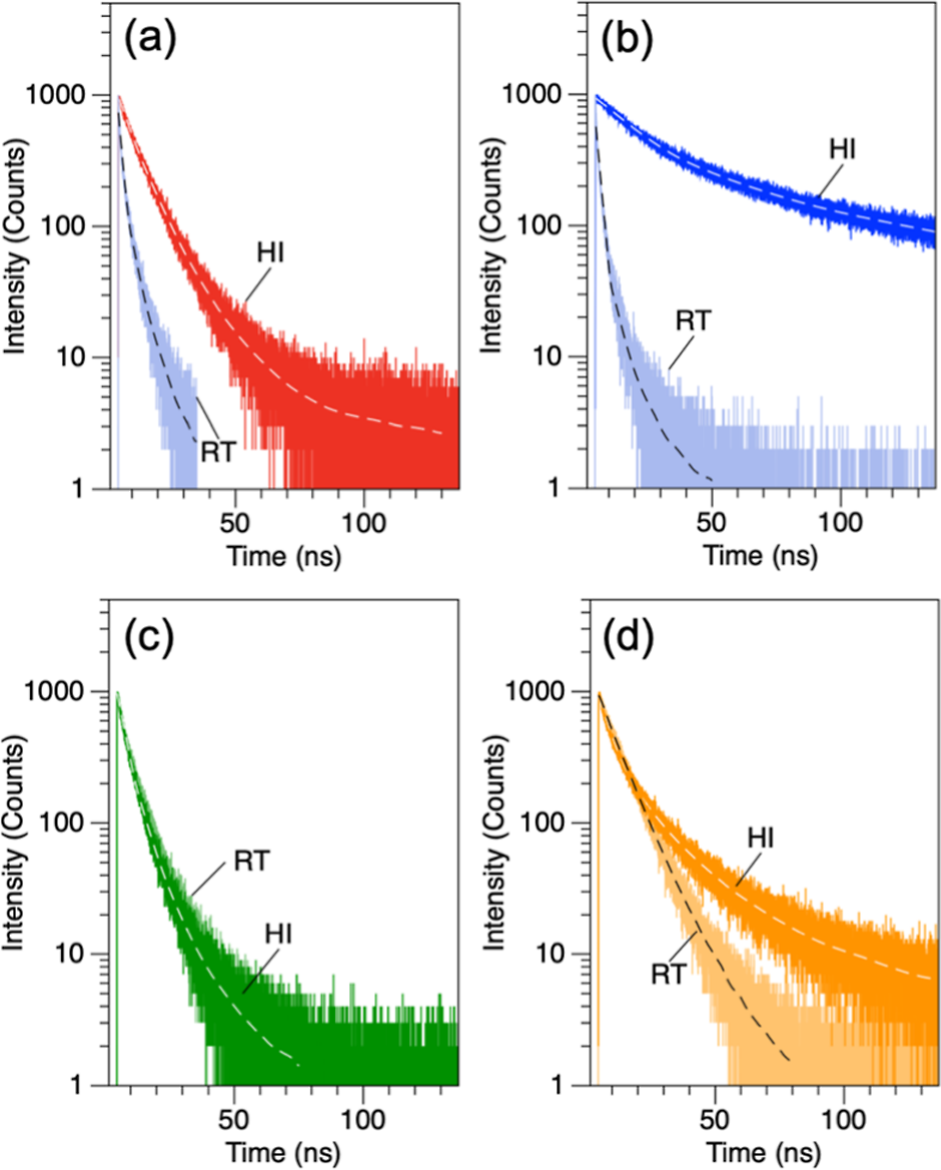
Representative TCSPC results comparing PL decay RT-CsPbBr_3_ (RT, *t* = 24 h) and HI-CsPbBr_3_ (HI, *t* = <1 min) in S = ODE (a), DBE (b), DOE (c), and DPE (d).

Taken together, these results show that solvent selection is important when considering the low-temperature synthesis of CsPbBr_3_ and that π-rich solvents, such as DBE and DPE play an important role in crystal growth and final morphology and optoelectronic properties, even in the presence of ligands like OAm and OAc. As described above, low-temperature synthesis,^51,52^ assembly,^86,87^ and flow chemistry^58,88^ routes are becoming increasingly important in the preparation of perovskites. Thus, the purposeful synthesis and detailed studies of future [PbBr_*n*_]^2–*n*^ plumbates are important, including those that utilize additional π-donating functionality, or a wider variety of ethereal solvents. In addition, mass spectroscopy has been used by researchers studying tin^73^ and lead iodide complexes^89^ previously, and a similar detailed approach correlating optical, ^207^Pb NMR, plumbate mass, and CsPbX_3_ product quality is part of our ongoing studies.

## Conclusion

In summary, the role that high-boiling-point solvents play in the formation of lead plumbate precursors used to prepare cesium lead halide nanocrystals was studied. Optical assays and ^207^Pb NMR show that the π-rich solvents dibenzyl ether and diphenyl ether coordinate strongly to Pb^2+^ and aid in the dissolution of PbBr_2_ to form [PbSBr_*n*_]^2–*n*^ plumbates. This coordination persists during the nucleation and growth steps, especially when a low-temperature synthesis is employed, such as the room temperature one-pot method described here. The results showed that growth could be tuned by the solvent type, even in the presence of ligands, but that its surfaces were highly defect rich, as illustrated by the fast PL decay. A parallel control study using the same [PbSBr_*n*_]^2–*n*^ precursors in a hot-injection synthesis showed less solvent effect, with similar morphologies, crystal structures, and PL lifetimes of the products across each solvent, suggesting that the elevated temperature when combined with the ligand environment is enough to overcome the S-to-Pb^2+^ π-coordination.

## Supplementary Material



## Notes

Analysis of PL decay fits, UV–vis transmission measurements for each solvent, BH analysis plots and fits, UV–vis of control plumbates, 207Pb NMR spectra of control plumbates, enlarged TEM micrograms with size analysis histograms, and powder XRD (PDF)
