# Supplementary material for: Investigating the Role π‑Rich Solvents Play in the Growth of Cesium Lead Bromide Nanocrystals
Source: ACS Nanosci Au. 2025 Oct 24;6(1):44–53. doi: 10.1021/acsnanoscienceau.5c00081 (PMC12921602; doi:10.1021/acsnanoscienceau.5c00081)
Supplement: Supplementary file 1 [file ng5c00081_si_001.pdf]

Investigating the Role π-rich Solvents Play in the Growth of Cesium Lead Bromide Nanocrystals

Tsung-Hsing Chiang[1], Deborah J. Kerwood[1], Abigail L. Stapf[1],
Mircea Cotlet[2], and Mathew M. Maye[1]*

[1]Department of Chemistry, Syracuse University, Syracuse, New York 13244, U.S.A.
[2]Center for Functional Nanomaterials, Brookhaven National Laboratory, Upton, NY, 11973

SUPPORTING INFORMATION

Table S1: TCSPC Fitting Results of PL decay shown in Figure 6.

	τ_1 (ns)	$A\tau_1$ (%)	τ_2 (ns)	$A\tau_2$ (%)	τ_{ave} (ns)
ODE-$CsPbBr_3$					
RT	1.04	65.44	4.49	34.56	2.23
HI	4.50	35.13	10.72	64.87	8.53
BE-$CsPbBr_3$					
RT	0.81	68.23	3.48	31.77	1.66
HI	18.46	66.72	94.05	33.28	43.62
OE-$CsPbBr_3$					
RT	2.67	39.76	7.69	60.24	5.69
HI	3.22	62.35	8.42	37.65	5.18
DPE-$CsPbBr_3$					
RT	8.81	100.00			8.81
HI	4.40	60.95	19.35	39.05	10.57

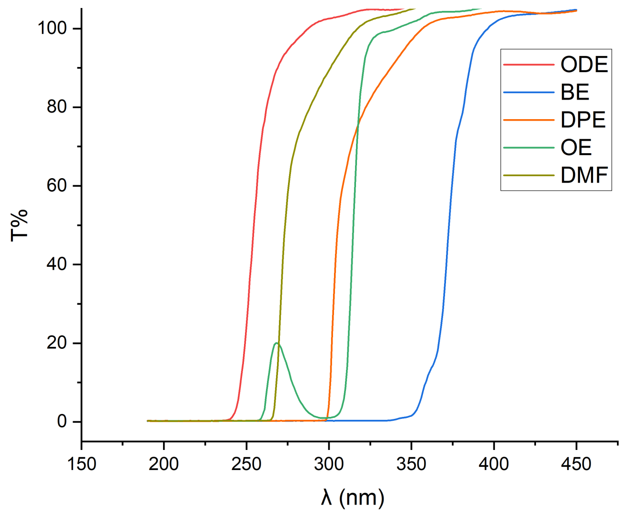

Figure S1. UV transmittance of ODE, DBE, DPE, DOE, and DMF solvents.

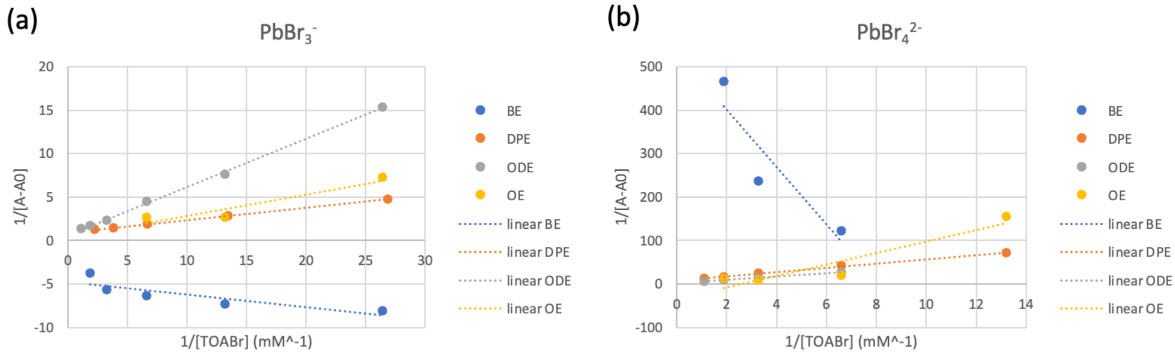

	Interception	Slope	Ka (mM^-1)	R^2
BE	-4.819	-0.144	n/a	0.7432
DPE	0.912	0.142	6.41490858	0.9995
OE	0.307	0.248	1.2363124	0.8925
ODE	0.622	0.523	1.19051262	0.9987

	Interception	Slope	Ka (mM^-1)	R^2
BE	533.180	-65.957	n/a	0.8313
DPE	7.286	4.921	1.48055431	0.9949
OE	-33.896	13.194	n/a	0.8813
ODE	1.066	3.855	0.27649088	0.9945

Figure S2. Benesi-Hildebrand plot from UV-vis data $[PbSBr_n]^{2-n}$ formed in S = BE (blue), DPE (orange), ODE (yellow), OE (gray) upon titration with TOABr and measured absorbance changes at 310 nm ($[PbBr_3]^-$) (a) and 360 nm ($[PbBr_4]^{2-}$) (b). Analysis tables shown.

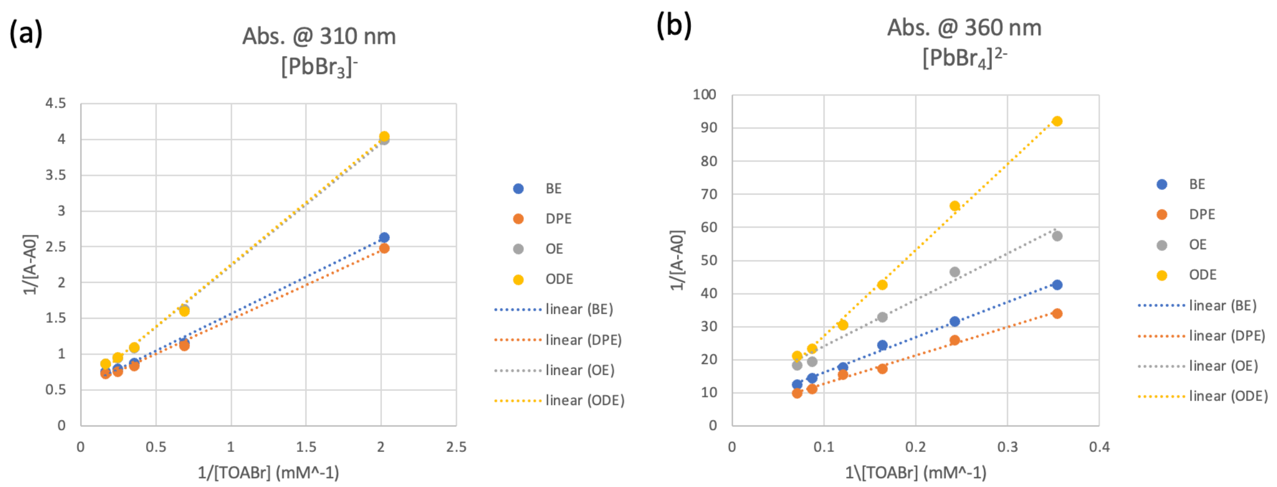

	interception	slope	Ka (mM^-1)	R^2
BE	0.534	1.028	0.520	0.9953
DPE	0.523	0.960	0.545	0.9968
OE	0.526	1.707	0.308	0.9985
ODE	0.515	1.734	0.297	0.9976

	interception	slope	Ka (mM^-1)	R^2
BE	5.145	109.430	0.047	0.9987
DPE	4.277	85.737	0.050	0.9925
OE	10.105	140.190	0.072	0.9712
ODE	1.425	259.430	0.005	0.9963

Figure S3. Benesi-Hildebrand plot from UV-vis data of $[Pb(DMF)_6]^{2+}$ with S= BE (blue), DPE (orange), ODE (yellow), OE (gray) first added, and then titrated with TOABr at 310 nm ($[PbBr_3]^-$) (a) and 360 nm ($[PbBr_4]^{2-}$) (b). Analysis tables shown.

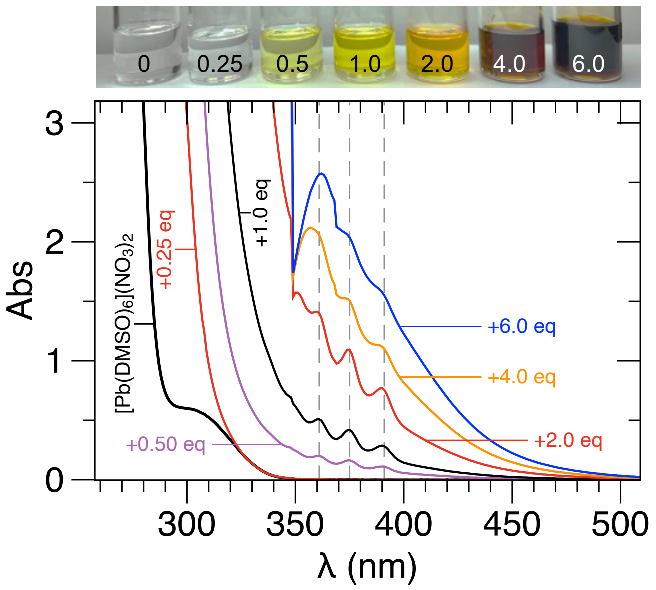

Figure S4: UV-vis of $PbNO_3$ dissolved in DMSO to form $[Pb(DMSO)_6](NO_3)_2$ with addition of 0.25, 0.50, 1.0, 2.0, 4.0, and 6.0 molar equivalents of HBr and reaction times of 12h. Vertical lines guide the eye at 361, 375, and 391 nm. Inset: Photograph of solutions with equivalents shown. Samples in photographs are ~1.0 M Pb^{2+} , while UV-vis utilized a 2mm pathlength quartz cuvette at concentration of 0.15M for 0 - 2.0 equivalents, and a lower 0.02 M concentrations for 4.0 and 6.0 equivalents due to the significant increase in extinction.

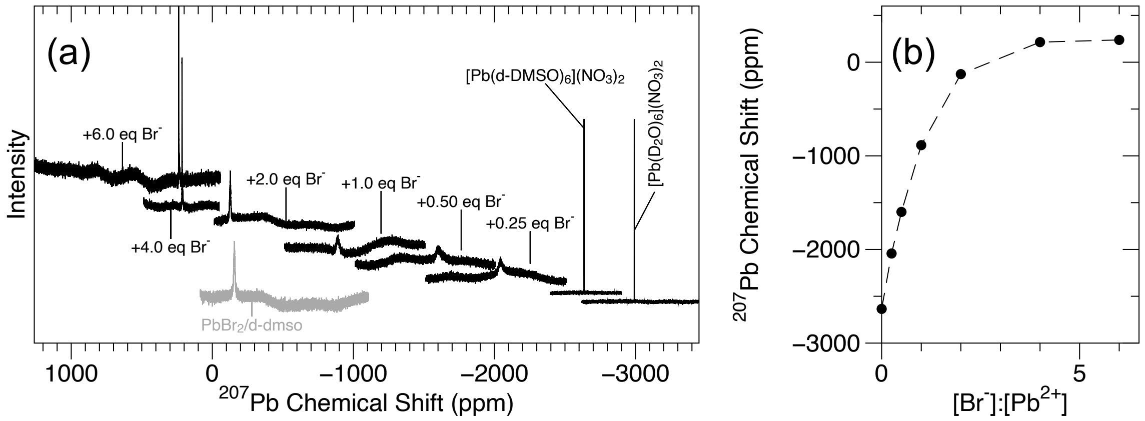

Figure S5: (a) A representative set of ^{207}Pb NMR results for the formation of $[Pb_xBr_y]^{2x-y}$ plumbates starting from [Pb(*d*-DMSO)$_6$]$(NO_3)_2$ (1.0 M) and formed upon the addition of molar equivalents (eq) of HBr. Each chemical shift (δ) position corresponds to a unique Pb environment. The $[Pb(D_2O)](NO_3)_2$ standard is shown for comparison, as is a control of $PbBr_2$ dissolved in *d*-DMSO (grey), confirming 2 eq Br- coordinating in those solutions. Data is off set vertically for clarity. (b) A plot of ^{207}Pb δ peak position as a function of $[Br^-]:[Pb^{2+}]$, dashed line to guide the eye. All samples at ~1.0 M Pb^{2+} in *d*-DMSO and measured after ~12h reaction times.

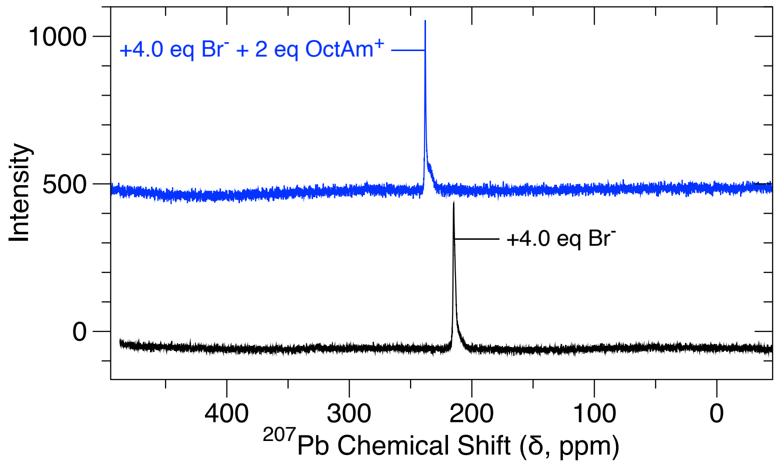

Figure S6: Comparison of ^{207}Pb NMR spectra corresponding to samples with 4 molar equivalents of HBr (black), and 4 molar equivalents of HBr plus 2 molar equivalents of octylamine (OctAm) (blue), which correspond to $[PbBr_4](NO_3)_2$ and $[PbBr_4](OctAm)_2$ plumbates. Both samples at 1.0M Pb^{2+} in *d*-DMSO solvents, after ~12h reaction times, data is off set vertically for clarity.

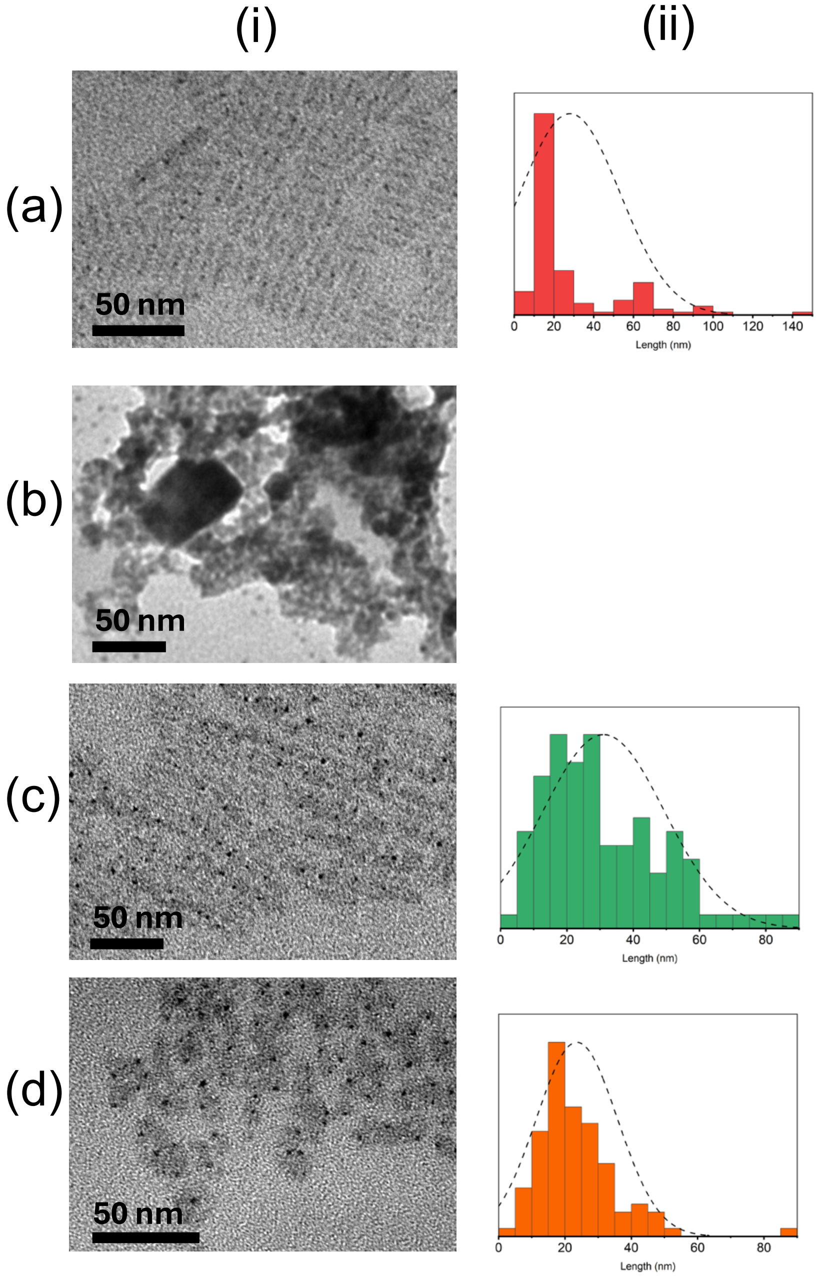

Figure S7. Enlarged TEM micrographs (i) and size distribution histograms (ii) from Figure 3 of RT-$CsPbBr_3$ products from t = 24h using S = ODE (a), DBE (b), DOE (c), and DPE (d). DBE histogram not shown due to poor morphology.

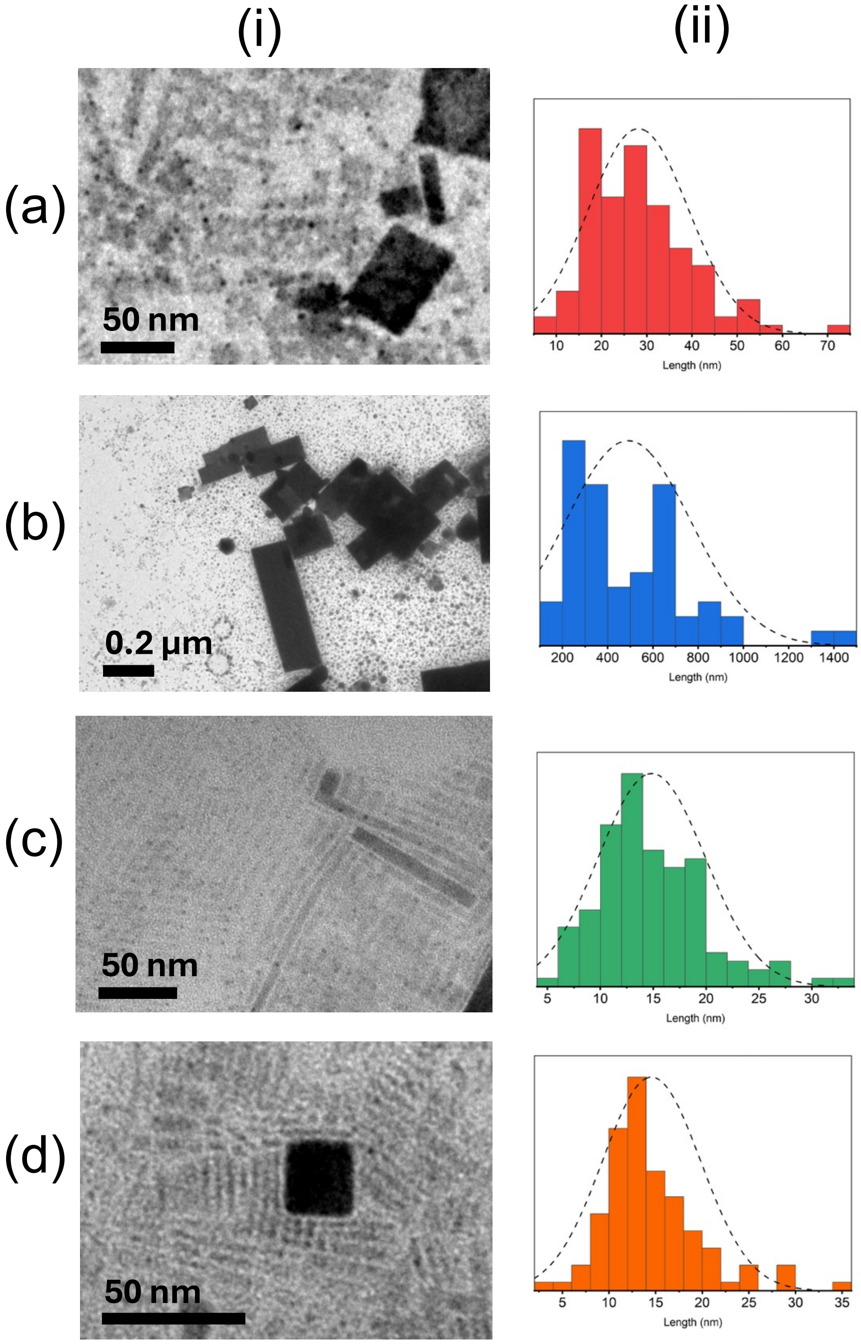

Figure S8. Enlarged TEM micrographs (i) and size distribution histograms (ii) from Figure 3 of RT-$CsPbBr_3$ products from t = 96h using S = ODE (a), DBE (b), DOE (c), and DPE (d).

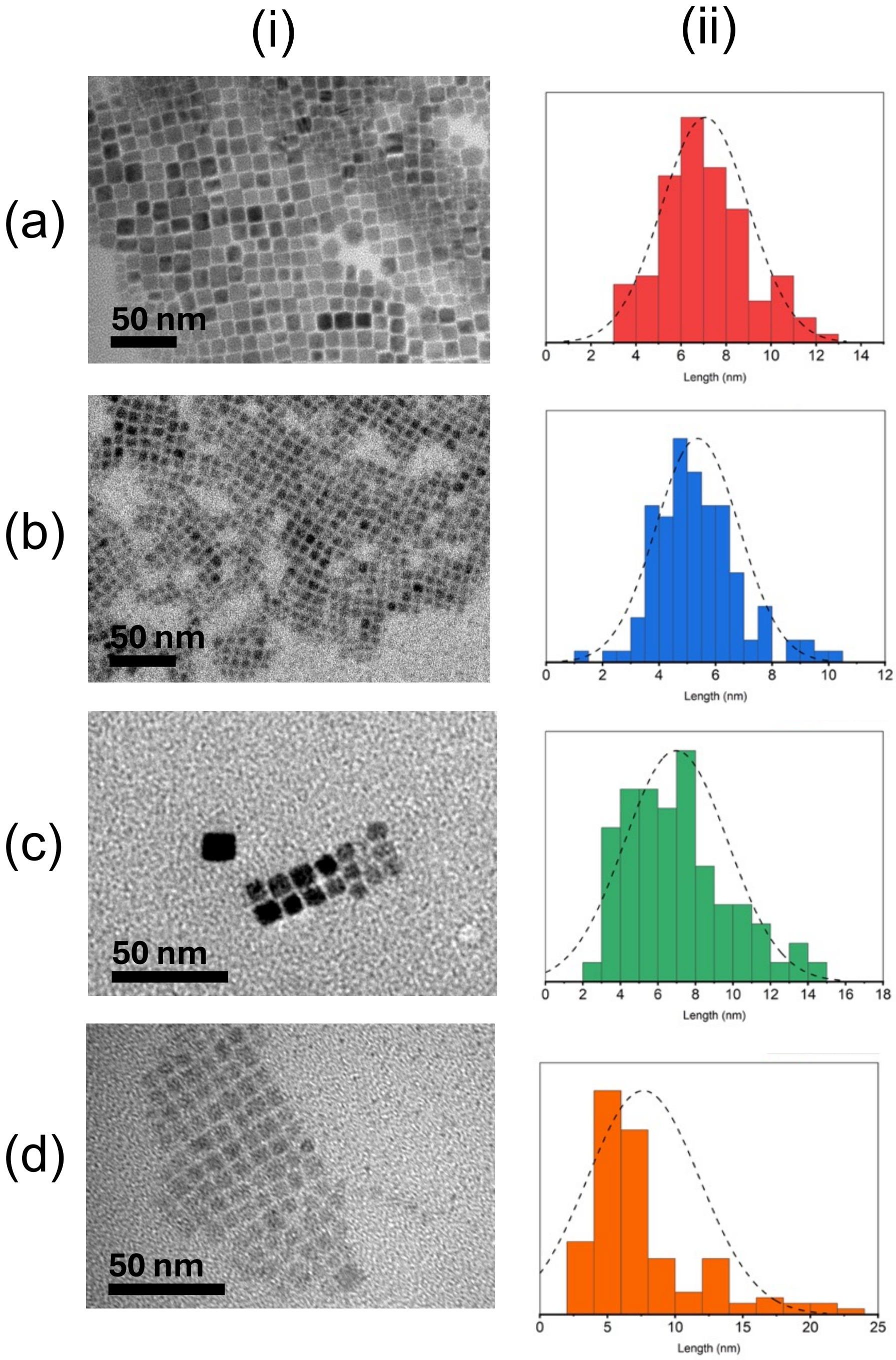

Figure S9. Enlarged TEM micrographs (i) and size distribution histograms (ii) from Figure 5 of HI-$CsPbBr_3$ products from t < 1 min using S = ODE (a), DBE (b), DOE (c), and DPE (d).

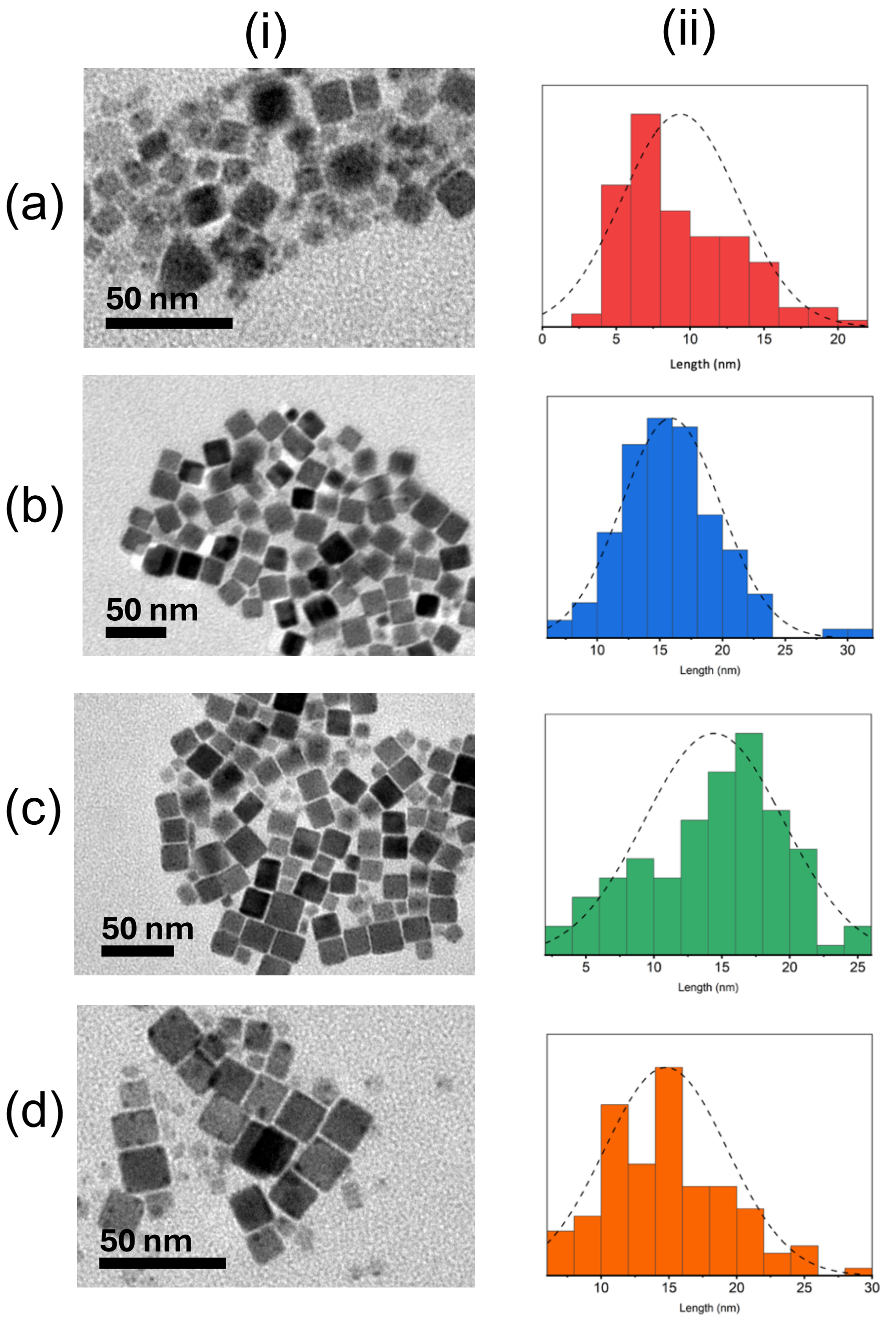

Figure S10. TEM micrographs (i) and size distribution histograms (ii) of HI-$CsPbBr_3$ products from t = 30 min using S = ODE (a), DBE (b), DOE (c), and DPE (d).

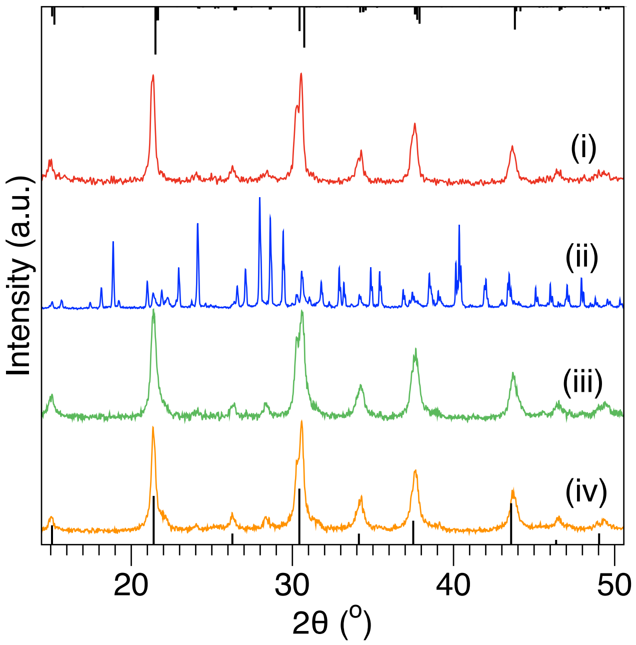

Figure S11. (a) Powder XRD of $CsPbBr_3$ prepared via RT at t = 96 (a) using ODE (i), DBE (ii), DOE (iii) and DPE (iv). Comparison $CsPbBr_3$ reference patterns for cubic (bottom, COD-1533063) and orthorhombic (top, COD-4510745) crystals shown.
